# Three-Dimensional Architecture and Biogenesis of Membrane Structures Associated with Plant Virus Replication

**DOI:** 10.3389/fpls.2018.00057

**Published:** 2018-01-30

**Authors:** Xuejiao Jin, Xiuling Cao, Xueting Wang, Jun Jiang, Juan Wan, Jean-François Laliberté, Yongliang Zhang

**Affiliations:** ^1^State Key Laboratory of Agro-Biotechnology and Ministry of Agriculture Key Laboratory of Soil Microbiology, College of Biological Sciences, China Agricultural University, Beijing, China; ^2^Institut National de la Recherche Scientifique—Institut Armand-Frappier, Laval, QC, Canada

**Keywords:** plant virus, viral replication factories, cellular remodeling, three-dimensional architecture, biogenesis

## Abstract

Positive-sense (+) RNA viruses represent the most abundant group of viruses and are dependent on the host cell machinery to replicate. One remarkable feature that occurs after (+) RNA virus entry into cells is the remodeling of host endomembranes, leading to the formation of viral replication factories. Recently, rapid progress in three-dimensional (3D) imaging technologies, such as electron tomography (ET) and focused ion beam-scanning electron microscopy (FIB-SEM), has enabled researchers to visualize the novel membrane structures induced by viruses at high resolution. These 3D imaging technologies provide new mechanistic insights into the viral infection cycle. In this review, we summarize the latest reports on the cellular remodeling that occurs during plant virus infection; in particular, we focus on studies that provide 3D architectural information on viral replication factories. We also outline the mechanisms underlying the formation of these membranous structures and discuss possible future research directions.

## Introduction

Positive-strand (+) RNA viruses induce extensive endomembrane reorganizations in the host cell to create a favorable microenvironment for their replication (Verchot, 2011; Romero-Brey and Bartenschlager, 2014). These remodeled membranous structures are thought to sequester virus replication processes away from host defense systems, such as RNA silencing. They are also thought to compartmentalize viral RNA, viral proteins, and the diverse host factors for high-efficiency synthesis of progeny RNAs (Schwartz et al., 2004; Novoa et al., 2005; Miller and Krijnse-Locker, 2008; Verchot, 2011).

Conventional transmission electron microscopy (TEM) provides only random or discontinuous pictures of cellular organelles (Baumeister, 2002) and thus may lead to misconceptions regarding cellular ultrastructure. For example, three-dimensional (3D) analysis of turnip mosaic virus (TuMV)-induced intracellular rearrangements revealed that the vesicle-like structures in two-dimensional (2D) TEM images are, in fact, tubules (Wan et al., 2015). To overcome the limitations of the random sectioning used in traditional TEM analysis, many novel 3D electron microscopy methods have been developed, including serial sectioning, electron tomography (ET), scanning transmission electron microscopy (STEM) tomography, serial block-face SEM (SBF-SEM), FIB-SEM, cryo-ET, and cryo-FIB-SEM (Romero-Brey and Bartenschlager, 2015). The development of these approaches has improved our understanding of the membrane rearrangements that occur during plant virus infection (Laliberté and Zheng, 2014; Risco et al., 2014; Harak and Lohmann, 2015; Fernández de Castro et al., 2017). This review provides a comprehensive overview of recent major progress in the 3D analysis of plant (+) RNA virus replication compartments and the mechanisms underlying their formation.

## 3D imaging techniques for the reconstruction of viral replication factories

Limited information is obtained from conventional TEM due to the small thickness of thin sections. Hence, serial sectioning is emerging as a method to overcome this problem. A long ribbon of serial sections is needed, and hundreds to thousands of micrographs are recorded from the serial sections. The micrographs are aligned and processed to a stack. 3D structures can be generated from the stack of images. Although it provides more information than conventional TEM, serial sectioning has several drawbacks. For example, this sectioning requires well-trained staff to obtain high-quality serial sections without losing a single section, and the discontinuities between two consecutive sections often create difficulties in the alignment of images.

In contrast to serial sectioning, more ultrastructural information can be obtained from thicker sections by electron tomography (ET). ET is a powerful technique that yields highly informative images of subcellular architecture in three dimensions using thick sections; it can show a wide range of subcellular structures present in 200–400-nm-thick resin sections at 3–8-nm resolution (Mastronarde, 1997; McIntosh et al., 2005). The images are collected using 200–400 kV intermediate voltage electron microscope (IVEM) with a eucentric specimen rod and a charge-coupled-device (CCD) camera (Donohoe et al., 2006). Images of resin sections with thicknesses of 200–400 nm are recorded from −60 (−70°) to +60 (+70°) in 1–2° angular increments to obtain an image stack containing 80–140 images. The generated images represent 1–4 nm thin slices (Donohoe et al., 2006). After recording the tilted images from the section, the grid is rotated 90°, and a second tilt series is acquired. Because tilting the specimen in single-axis tomography provides the so-called missing-wedge information, dual-axis ET analysis can complement the missed high-tilt projection data in single-axis ET. The obtained image stack can be processed and surface-rendered by 3D image processing software to obtain a virtual 3D volume; the method thus provides detailed information and contributes to our understanding of the overall 3D architecture of the cell (Mastronarde, 1997; McIntosh et al., 2005). This technique has been widely used to reveal the important features of cellular organelles (Otegui et al., 2001, 2006; Otegui and Staehelin, 2004; Seguí-Simarro et al., 2004; Shimoni et al., 2005; Austin et al., 2006; Leitz et al., 2009; Kang et al., 2011; Kowalewska et al., 2016). However, due to the thickness limitation, electron tomography based on transmission electron microscopy is not applicable to structures with large volumes, such as mitochondria and chloroplasts. Data collection from serial thick sections (serial ET) can solve this problem; however, similar to serial sectioning, this approach is time-consuming, and the preparation of high-quality serial sections requires highly skilled staff. The limited range of tilting angles also makes the recording of overall structural data impossible.

Other powerful 3D imaging technologies, such as SBF-SEM and FIB-SEM, which is also known as focused ion beam-field emission scanning electron microscopy (FIB-FESEM), have the additional advantage of making it possible to reconstruct large-volume structures and can be used to overcome the thickness limitations of ET. With SBF-SEM and FIB-SEM, sectioning is performed automatically inside the SEM microscope using a diamond knife or a focused ion beam. After sectioning or milling, the freshly exposed block face is tilted vertically toward the electron beam for imaging (Romero-Brey and Bartenschlager, 2015). Hence, SBF/FIB-SEM comprehensively utilizes diamond knife/ion and electron beams to “slice and view” a set of images that can be used to generate 3D volumes. SBF-SEM and FIB-SEM are more suitable than ET for the 3D reconstruction of mesoscale structures, providing fine structural details while considering the links between the target structures (Marko et al., 2007; Drobne et al., 2008; Rigort et al., 2012; Kizilyaprak et al., 2014a,b; Rigort and Plitzko, 2015). In SBF-SEM and FIB-SEM, serial block-face imaging, sectioning and imaging of the sample are automatic; the use of these methods thus avoids many of the problems associated with manual sectioning. The numbers of the images depend on the Z-depth of desired volume and the Z-resolution is defined by the thickness of the slices. In comparison to SBF-SEM, the slice thickness is developed to 3 nm in FIB-SEM, whereas the minimum slice thickness is about 25 nm in SBF-SEM with optimal specimen. Hence, the main advantages of FIB-SEM over SBF-SEM are a significant improvement in z-axis resolution. Moreover, FIB-SEM enables targeting a small region of interest without destroying the remainder of the block face, allowing the rest of the block face to be used for subsequent resampling (Arkill et al., 2014; Peddie and Collinson, 2014). Hence, FIB-SEM has been widely used in the study of structures in virus-infected cells (Bennett et al., 2009; Felts et al., 2010; Do et al., 2014; Gómezaix et al., 2015). However, because the block face of the sample is destroyed during either FIB-SEM or SBF-SEM analyses, these methods cannot be used when it is necessary to retain the samples.

In recent years, cryo-ET has become a rapidly developing technology that offers high resolution. Unlike cryo-EM, which relies on symmetrical targets to generate 3D images and has been used for several decades to image viral structures, cryo-ET provides a way to image irregular structures in samples prepared by cryo-methods, and it can maintain samples in a considerably more native state than can be achieved using chemical fixation. Additionally, with recent advances in camera technology, cryo-ET now offers resolution at the molecular level. The use of cryo-ET in the visualization of virus replication factories has revealed several previously unrecognized features of these structures during TEM and ET analyses. However, the maximal thickness of the samples used in cryo-ET is 1 μm, which restricts its application in resolving structures with large volumes (Cyrklaff et al., 2007; Ertel et al., 2017). At present, ET and FIB-SEM are the most widely used methods in the study of the structures of animal and plant virus replication factories (Laliberté and Zheng, 2014; Risco et al., 2014; Harak and Lohmann, 2015; Fernández de Castro et al., 2017). The characteristics and advantages of various 3D electron microscopic techniques have been accurately summarized in recent reviews, and the protocols used for sample preparation can be found in specialized publications (Kuo, 2007; Zárský and Cvrčková, 2014; Romero-Brey and Bartenschlager, 2015).

## 3D architecture of plant (+) RNA virus replication factories

Viral replication factories are derived from a variety of organelles, and the selection of organelles for building the replication factories depends on the virus. These organelles include the endoplasmic reticulum (ER), peroxisomes, mitochondria, chloroplasts, and tonoplasts (Verchot, 2011). Interestingly, the membranes responsible for the formation of replication factories of a virus may change, and viruses can use multiple organelles for replication (Xu and Nagy, 2014). For example, tomato bushy stunt virus (TBSV) utilizes peroxisomes to establish replication compartments. However, in yeast mutants in which peroxisome biogenesis is defective, TBSV replication sites are derived from the ER, which provides an optional subcellular membrane for virus replication (Jonczyk et al., 2007; Chuang et al., 2014). Similarly, when the targeting of flock house virus (FHV) replication protein A was changed from mitochondria to the ER, protein A was still capable of inducing membrane alterations and supporting FHV replication (Miller et al., 2003), suggesting that there is flexibility in the selection of organelles for building viral replication factories under different conditions. Despite their origins in different organelles, the 3D structures of numerous (+) RNA virus replication factories reveal morphological similarities among different virus families. These similarities are manifested by two morphotypes. One morphotype is characterized by the formation of invaginated spherules with neck-like channels that connect the interior of the spherule to the cytoplasm. The second morphotype is characterized by the presence of single and/or double-membrane vesicles (SMVs or DMVs) that are formed by the remodeling of endomembranes (den Boon et al., 2010; Paul and Bartenschlager, 2013). It is worth noting that a new type of structure termed “appressed double-membrane layers” appeared when the expression ratio of brome mosaic virus (BMV) replication proteins was altered; these structures also supported BMV replication (Schwartz et al., 2004).

Since Kopek et al. reported the first 3D architecture of the membrane-bound (+) RNA viral replication compartments in FHV-infected *Drosophila* cells (Kopek et al., 2007), several 3D models of cellular remodeling during plant virus infection have been characterized. These are summarized in the following subsections.

### Beet black scorch virus (BBSV)

**Figure 1:**
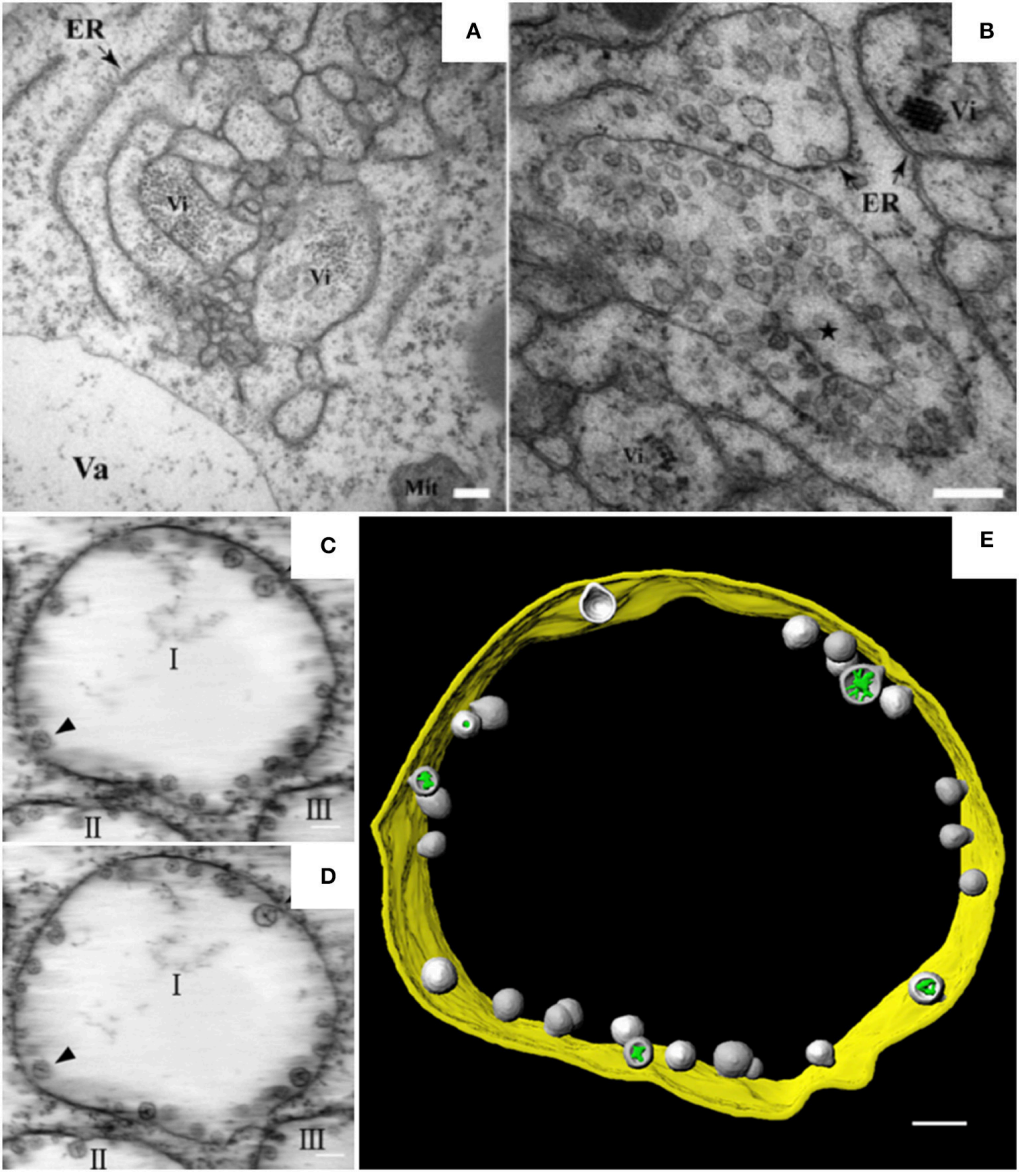
BBSV remodels ER membranes and 3D model of BBSV-induced vesicle packets. **(A)** BBSV infection led to ER aggregation and vesiculation. **(B)** Vesicle packets were observed in the aggregates of branched ER cisternae (star). **(C,D)** Slices from the electron tomogram of BBSV-induced vesicle packets and spherules derived from ER. Arrowheads indicate the same spherules in different slices. The spherules are connected to the outer ER membrane. **(E)** Three-dimensional model of BBSV-induced vesicle packets and spherules derived from ER. Vi, virus particles or virus crystals; Va, vacuole; gold, vesicle packet derived from ER outer membrane; gray, spherules; green, fibrillar materials within the spherules. This figure is adapted with permission from Cao et al. (2015) (© 2015 by the American Society for Microbiology).

Alpha- and beta-necroviruses usually undergo replication on membranes derived from the ER or from tonoplasts (Kassanis et al., 1970; Appiano and Redolfi, 1993; Lot et al., 1996). BBSV, a *Betanecrovirus* in the *Tombusviridae* family, has a single-stranded RNA genome of positive polarity (Cao et al., 2002; Yuan et al., 2006). The genomic RNA of BBSV contains six open reading frames. Two of the encoded proteins are p23 and p82. p23 is an auxiliary replication protein, and p82 is a read-through product of p23 that possesses RNA-dependent RNA polymerase activity. Both proteins localize to the ER and are essential for the assembly of virus replication factories (Cao et al., 2015). In BBSV-infected *Nicotiana benthamiana* leaves, the ER aggregates to form punctate structures that can be observed by confocal laser scanning microscopy (CLSM) (Cao et al., 2015). These punctate structures or aggregates are thought to be associated with viral replication. TEM analysis further revealed the dilation, proliferation, and convolution of ER membranes (Figure 1A) and the formation of vesicle packets (VPs) along the ER (Figure 1B) or in the perinuclear cytoplasmic region (Cao et al., 2015). These rearranged membranous structures are likely the punctate structures observed by CLSM. In 2015, Cao et al. used ET to develop the first 3D model of an ER-associated replication compartment of a plant (+) RNA virus (Cao et al., 2015). These researchers' proposed 3D model shows the presence of one to several hundreds of spherules 50–70 nm in diameter in the interior of the VPs (Figures 1C–E). Viral double-stranded RNA (dsRNA), which is a genome replicative intermediate, and the replication protein p23 are both localized within the spherules, suggesting that these structures are sites of BBSV replication (Cao et al., 2015). BBSV spherules are arranged along the VP membranes, and most of them have a narrow neck connecting the spherule interior to the cytoplasm (Figures 1C–E), suggesting that they are formed from the invagination of ER membranes (Cao et al., 2015). Three animal viruses, dengue virus (DENV), Zika virus (ZIKV), and West Nile virus (WNV), all of which replicate on ER-derived membranes, also induce the formation of convoluted membranes (CMs) and/or VPs, and their replication occurs within membrane invaginations that originate from the ER and have openings to the cytosol (Welsch et al., 2009; Gillespie et al., 2010; Cortese et al., 2017). Surprisingly, in severe acute respiratory syndrome coronavirus (SARS-CoV)-infected cells, the inner vesicles in VPs show no pore connections to the outside (Knoops et al., 2008). Similar invaginations have been reported in the replication factories of other animal viruses such as FHV and Semliki Forest virus (SFV), although the replication factories of those viruses are derived from the mitochondrial membrane and the plasma membrane, respectively (Kopek et al., 2007; Kallio et al., 2013). However, pores interconnecting individual vesicles within the VPs of WNV are not observed in BBSV-induced spherules (Gillespie et al., 2010). Intriguingly, the VPs induced by BBSV are connected to each other by tubule-like structures 15–30 nm in diameter; such structures are rarely observed in other virus-induced membrane rearrangements (Cao et al., 2015). In addition, the putative viral RNAs of BBSV are observed as fibrillar materials with diverse morphologies, and these materials differ from those of WNV in their spatial distribution (Cao et al., 2015). In summary, it is obvious that the ER is commonly hijacked as a platform for the formation of viral replication bodies, although the morphologies of the replication sites differ (Romero-Brey and Bartenschlager, 2016).

### Tomato bushy stunt virus (TBSV)

**Figure 2:**
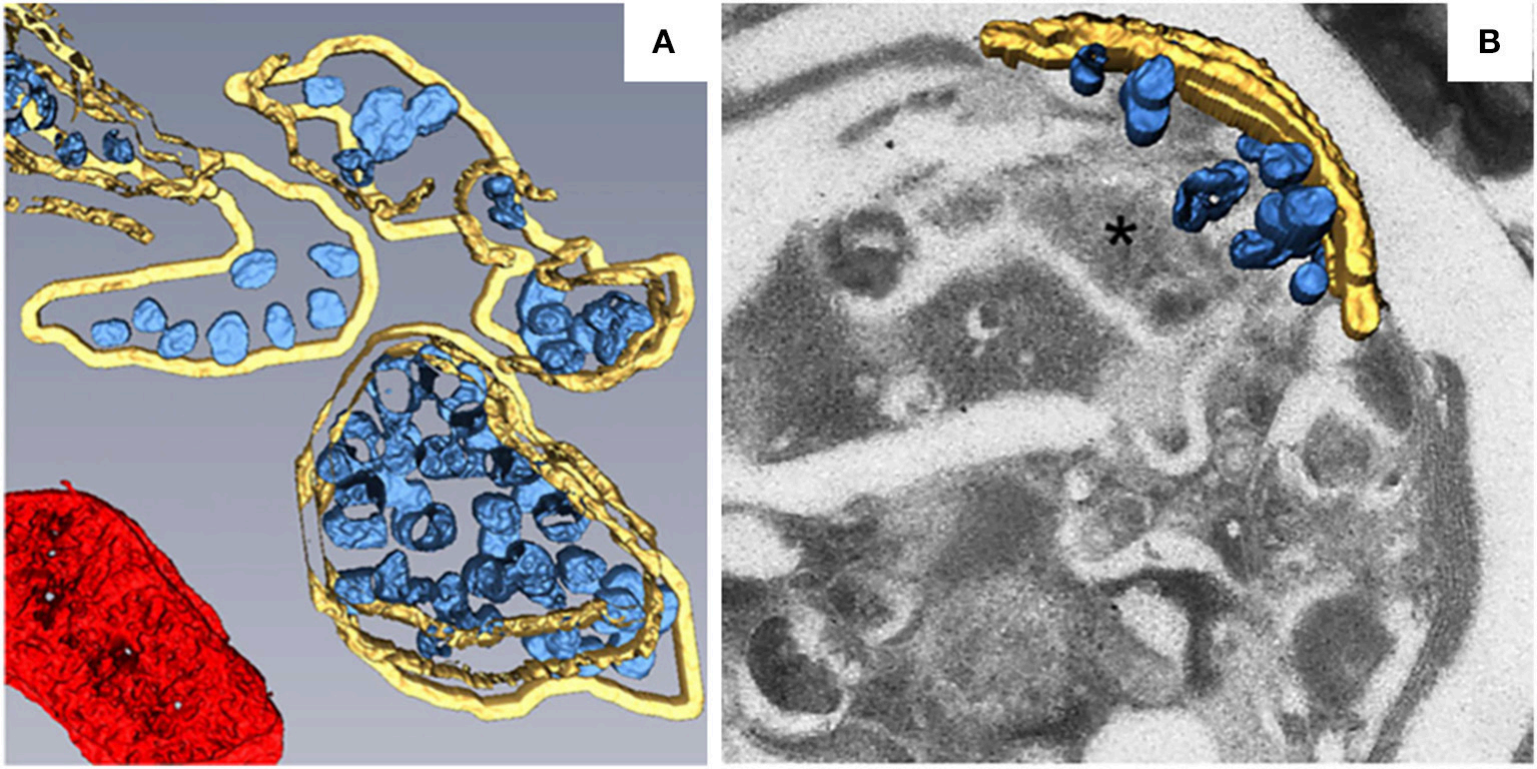
3D reconstruction of the TBSV replication platform in yeast. **(A)** 3D model of the TBSV replication platform in wild-type yeast cells characterized by peroxisome peripheral MVBs. Yellow, peripheral MVB membranes; blue, vesicle-like spherules; red, a mitochondrion. **(B)** 3D model of TBSV replication platform in Δpah1 yeast cells characterized by a large membrane compartment (asterisk) containing MVB-like structures with spherules and stacked ER. Yellow, stacked ER membranes; blue, spherules. This figure is adapted with permission from Fernández de Castro et al. (2017) (© 2017 by the Company of Biologists).

Most of the plant viruses that have been reported to build their replication factories on peroxisomes belong to the family *Tombusviridae*. TBSV is a well-studied (+) RNA virus in the genus *Tombusvirus* in the family *Tombusviridae*. During TBSV infection, the peroxisomal boundary membranes become progressively vesicular, leading to the formation of doughnut- or C-shaped multivesicular bodies (MVBs), the interiors of which contain many single-membrane vesicle-like structures 80–150 nm in diameter. These vesicles appear to be connected to the MVB boundary membrane through a neck (Martelli et al., 1988; McCartney et al., 2005), and they provide the sites for TBSV replication (Appiano et al., 1984; Martelli et al., 1988; McCartney et al., 2005; Barajas et al., 2014a). MVBs are frequently observed in close association with the ER. Since some membranous materials used in the vesiculation of peroxisomes are derived from the ER (Martelli et al., 1988), this may explain TBSV's ability to utilize the ER for VRC assembly in the absence of peroxisomes (Jonczyk et al., 2007; Chuang et al., 2014). To better characterize the fine structure of TBSV replication factories and the distribution of viral replication proteins, metal-tagging transmission electron microscopy (METTEM) and 3D molecular mapping were used to reconstruct the TBSV replication platform in wild-type yeast (Figure 2A) and in a yeast strain in which a gene encoding phosphatidate phosphatase (*pah1*) was deleted (Figure 2B; Fernández de Castro et al., 2017). The absence of *pah1* in yeast induces the proliferation and expansion of ER membranes. Interestingly, TBSV replicates more efficiently in Δ*pah1* mutant yeast cells than in wild-type cells (Csaki and Reue, 2010; Chuang et al., 2014). 3D imaging shows that whereas the MVBs in TBSV-infected wild-type yeast cells consist of large numbers of spherules that are connected to the MVB boundary membrane, in Δ*pah1* yeast cells, these vesicles are surrounded by and connected to the expanded ER membrane stacks instead of the peroxisomal MVB boundary membrane (Figures 2A,B). The architecture of the replication factories of TBSV in wild-type yeast is thus of the invaginated spherule type. METTEM analysis revealed that p33 is localized in both the MVBs and the ER, whereas viral dsRNA was only concentrated in the MVBs, suggesting that active RNA replication occurs in MVBs (Fernández de Castro et al., 2017). Because the biogenesis of peroxisomes involves ER membranes (Tabak et al., 2013), MVBs in Δ*pah1* yeast are thought to be nascent peroxisomes that are defective for release from the ER (Fernández de Castro et al., 2017). In Δ*pah1* yeast, the attachment and connections of these vesicles to the ER creates a network that resembles the single network of interconnected membranes derived from the ER that is present in cells infected with flaviviruses, coronaviruses, or arteriviruses. The interconnected membranes ensure the rapid transport of translated viral proteins to the replication sites, suggesting a close link between viral protein translation and processing (Risco et al., 2014).

### Melon necrotic spot virus (MNSV)

**Figure 3:**
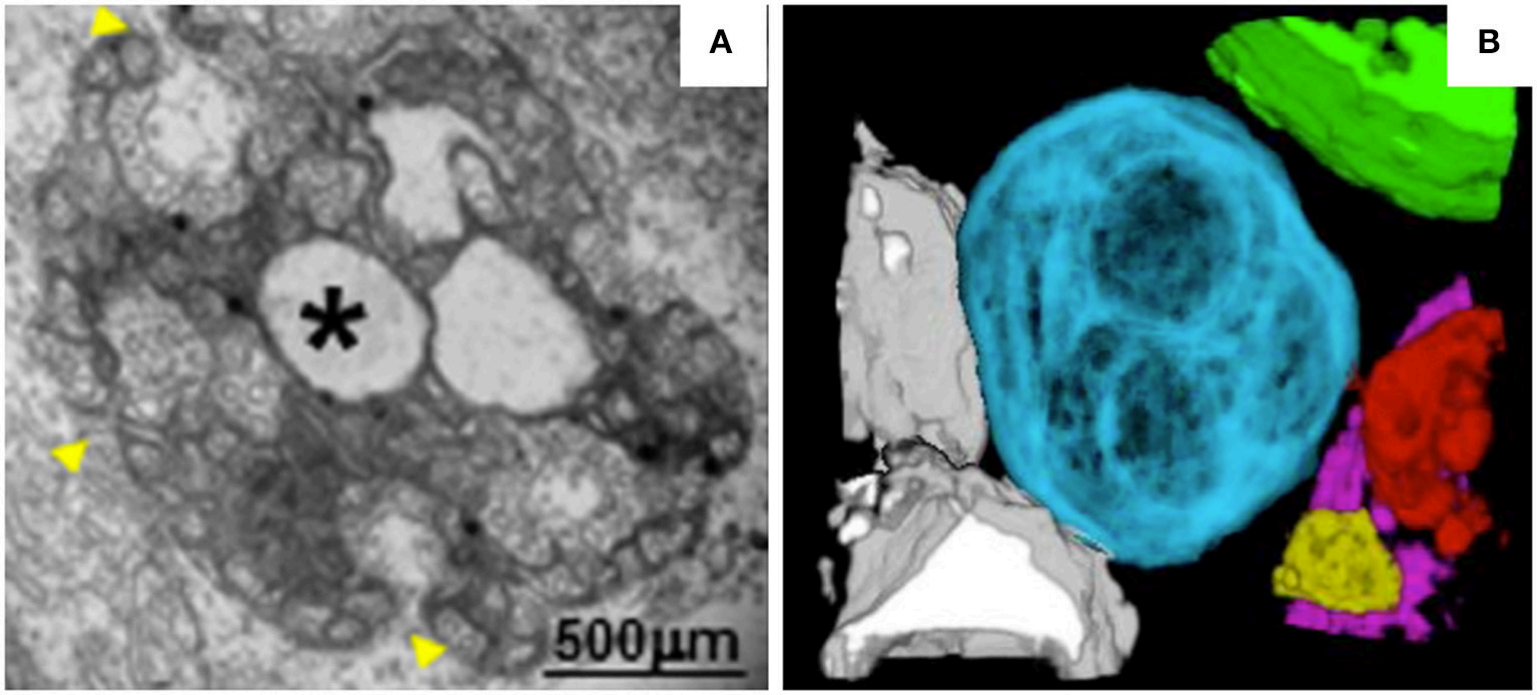
TEM analysis and 3D reconstruction of MNSV-induced altered mitochondria. **(A)** TEM image showing altered mitochondria. Numerous vesicles were observed on the external surface as well as internal large invaginations and internal dilations (star), or both. Yellow arrowheads indicate the pores connecting the lumen of the dilation to the surrounding cytoplasm. **(B)** 3D model of MNSV-induced altered mitochondria with large dilations inside analyzed by FIB-FESEM. Connections between different mitochondria as well as with lipid bodies are observed. Blue, yellow, red, and purple indicate altered mitochondria; chloroplasts are shown in green and lipid bodies in gray. This figure is adapted with permission from Gómezaix et al. (2015) (© 2015 by the American Phytopathological Society).

MNSV belongs to the genus *Carmovirus* in the family *Tombusviridae*. Like TBSV and cymbidium ringspot virus (CymRSV), MNSV induces the formation of MVBs (Russo et al., 1983; McCartney et al., 2005; Fernández de Castro et al., 2017). However, unlike the MVBs of TBSV, the MVBs of MNSV originate from mitochondrial membranes (Burgyan et al., 1996). In MNSV-infected cells, mitochondrial structure is dramatically altered. For example, the mitochondrial matrix decreases in size or enlarges, forming dilations, and the periphery of the mitochondrial membrane becomes vesicular (Figure 3A). Single-membrane vesicles 45–50 nm in diameter are formed along the mitochondrial membranes and around the large dilations inside the mitochondria. These vesicles appear to be connected to the cytoplasm or to the dilated lumen through neck-like structures (Gómezaix et al., 2015), as has been shown for FHV; viral infection thus induces mitochondria-derived invaginations with necks that are oriented toward the cytosol. Immunolocalization of MNSV (+) RNA, dsRNA, and CP revealed that these components are present in the large dilations of the altered mitochondria, suggesting that active MNSV replication occurs in the altered mitochondria and possibly in the numerous 50-nm vesicles (Gómezaix et al., 2015). 3D reconstruction of the mitochondria in MNSV-infected cells using FIB-FESEM reveals that these altered organelles possess many inner dilations that may be connected with each other (Figure 3B). These large dilations also have pores facing toward the outside cytoplasm that may be responsible for the exchange of materials required for viral replication. Although the vesiculation of the boundary membranes of replication organelles is similar to the vesiculation that occurs in many other viral infections, the presence of surrounding vesicles that are connected to the large dilations within mitochondria is a distinct characteristic of MNSV infection. Moreover, the 3D structure of the replication organelles of MNSV reveals a striking similarity of MNSV replication factories to those of TBSV, in which the altered mitochondria are always associated with the ER, lipid bodies, or lipid droplets (LDs) (Figure 3B; Martin and Parton, 2005) and in close proximity to plasmodesmata. LDs are also observed in close proximity to the replication factories of numerous animal viruses (Miyanari et al., 2007; Fernández de Castro et al., 2014). The membranous structures induced by hepatitis C virus (HCV) are primarily derived from the ER but also contain membranes derived from other compartments such as LDs that are important for HCV assembly. Hence, the connections between altered mitochondria and lipid bodies revealed by 3D reconstruction suggest a possible role of lipid bodies in MNSV replication, assembly, and other processes associated with viral infection.

### Turnip mosaic virus (TuMV)

**Figure 4:**
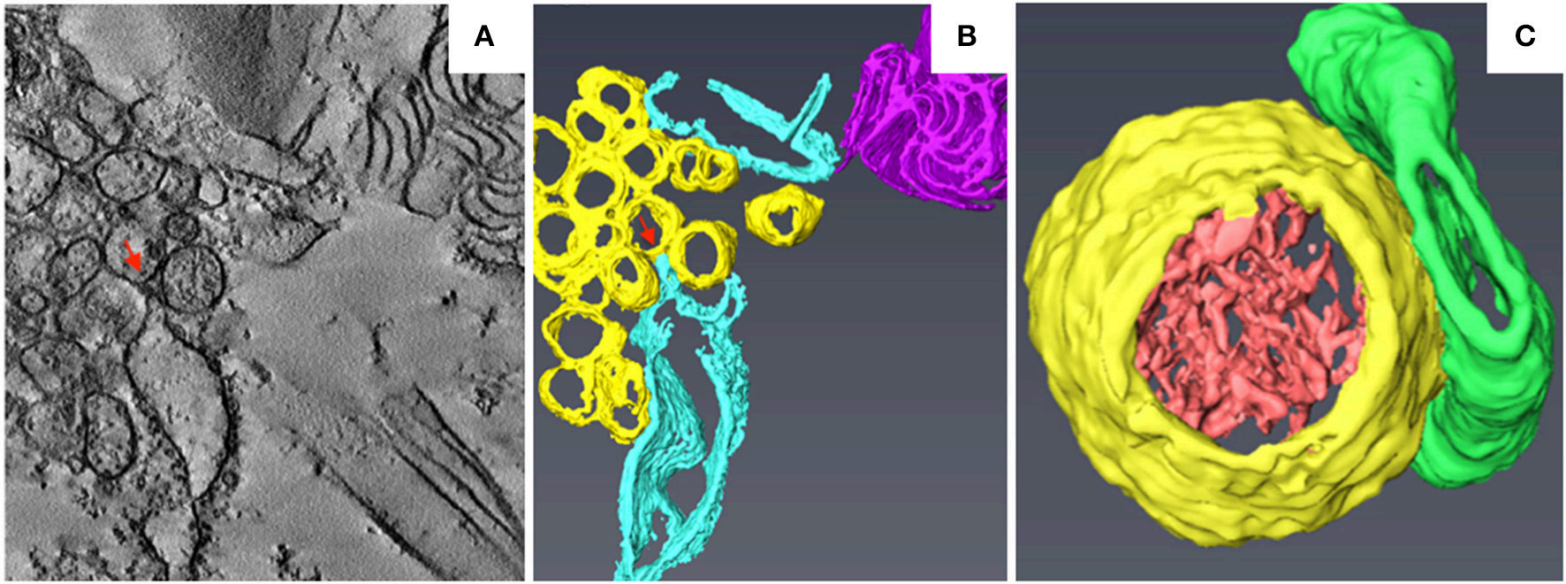
3D reconstruction of TuMV-induced SMTs at midstage of infection. **(A)** Tomogram slice from TuMV-infected vascular parenchymal cell. SMVL structures are seen in close proximity to dilated rER. **(B)** 3D surface rendering of TuMV-induced SMTs that connect with the rough ER. Yellow, SMTs; sky blue, rough ER; magenta, cytoplasmic inclusion body; red arrows, connection between the rough ER membrane and an SMT. **(C)** 3D model of the SMT with fibrillar materials inside. Yellow, SMTs; light red, electron-dense materials; green, intermediate tubular structures. This figure is adapted with permission from Wan et al. (2015) (© 2015 by the American Society for Microbiology).

TuMV is a (+) RNA virus belonging to the genus *Potyvirus* in the family *Potyviridae*. TuMV replicates in ER-derived vesicles that are formed at endoplasmic reticulum exit sites (ERES), as evidenced by the accumulation of viral RNAs and replication-related proteins within these vesicles (Wei and Wang, 2008; Grangeon et al., 2012). Unlike the BBSV- and BMV-induced membrane invaginations of the ER (Schwartz et al., 2002; Cao et al., 2015), the ER-derived replicative vesicles of TuMV are motile and align with microfilaments (Cotton et al., 2009). A time-course TEM analysis of TuMV-infected cells revealed the sequential formation of ER-derived membranous structures characterized by CMs, single-membrane vesicle-like structures (SMVLs) (Figure 4A), double-membrane vesicle-like structures (DMVLs), and electron-dense bodies (Wan et al., 2015). The CMs observed in TuMV-infected cells are similar to those induced by DENV, ZIKV, and SARS-CoV, which have been proposed to be the sites of viral polyprotein synthesis and processing (Knoops et al., 2008; Welsch et al., 2009; Cortese et al., 2017). 3D reconstruction of these membranous structures using ET indicated that the SMVLs and DMVLs observed in 2D TEM are in fact tubules (Figure 4B; Wan et al., 2015). Immunoelectron microscopic analyses of replication-related proteins showed specific labeling of membrane aggregates, and dsRNA was found to be specifically enriched in the single-membrane vesicle tubules (SMTs), suggesting that the SMTs are the true sites at which TuMV replication occurs. SMTs are usually regularly arranged and associated with the rough ER (Figure 4B), and the 3D morphology of the electron-dense fibrillar material inside the SMTs is similar to that within BBSV-induced replicative spherules (Figure 4C; Cao et al., 2015). As the infection proceeds, the SMTs are transformed into double-membrane vesicle tubules (DMTs) and intermediate tubular structures (Figure 4C). Interestingly, the SMTs and DMTs are similar to the membranous structures induced by coxsackievirus B3 and poliovirus, which are characterized by a transition from SMLs to DMLs during the course of virus infection. Likewise, in TuMV-, coxsackievirus B3-, and poliovirus-infected cells, DMLs with materials inside are formed by apposition, enwrapping, or fusion of SMTs (Limpens et al., 2011; Belov et al., 2012). Electron-dense bodies associated with virus-particle-like filament bundles are also formed at the late stage of infection, suggesting that virus particle assembly occurs there. At the final stages of infection, virus particles are found in the vacuoles, a location that might be favorable for aphid transmission of TuMV (Bak et al., 2017). ET analysis of TuMV-induced membranous structures at various time points during infection has thus made it possible to obtain a comprehensive overview of the changes that occur in endomembranes during the course of infection.

### Barley stripe mosaic virus (BSMV)

**Figure 5:**
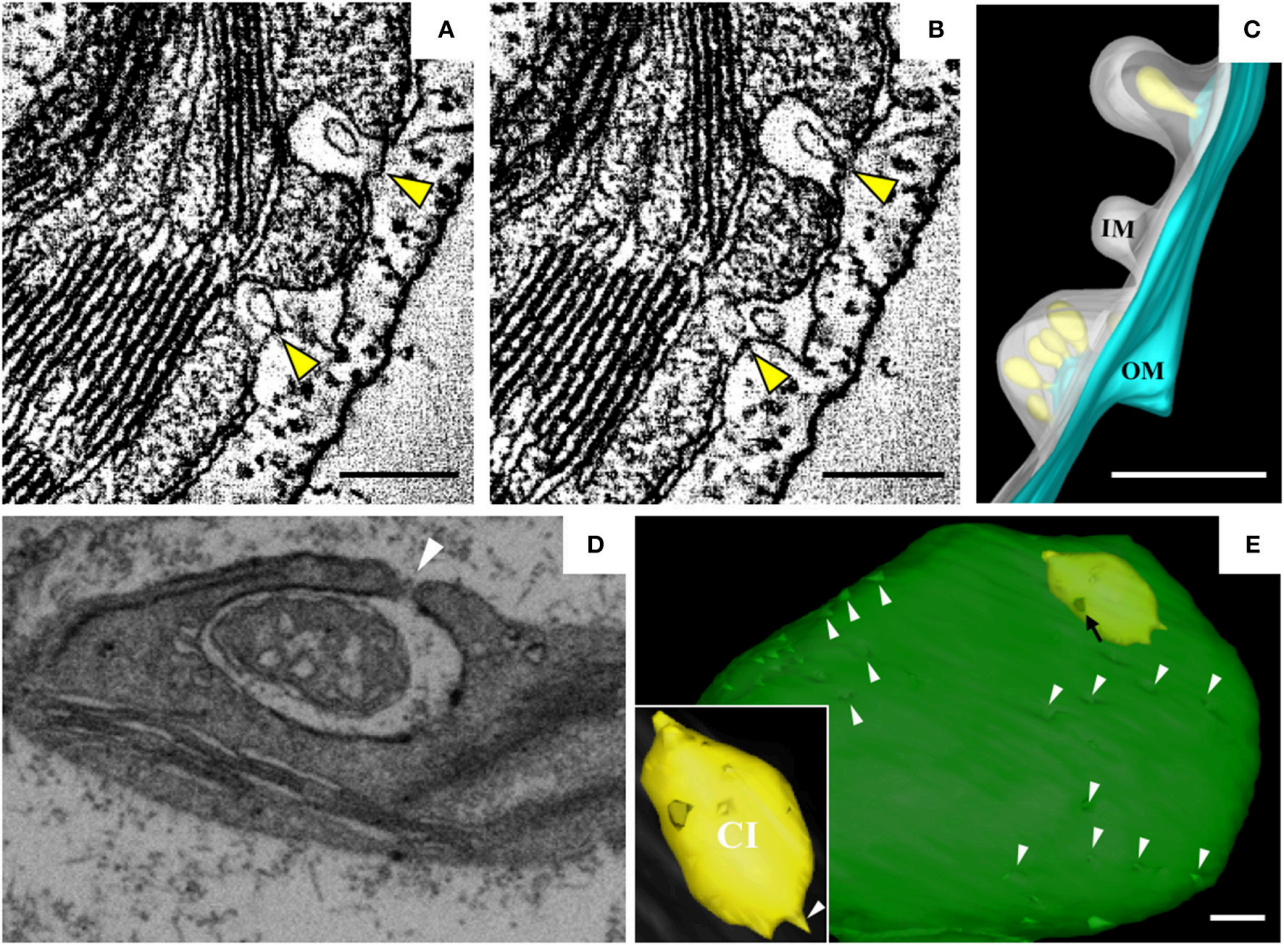
BSMV-induced chloroplast membrane rearrangement and 3D model of altered chloroplast membranes. **(A,B)** Tomogram slices of altered chloroplast membranes from leaves of BSMV-infected *N. benthamiana*. The arrowheads indicate the same spherules in different slices. **(C)** 3D model of remodeled chloroplast membranes induced by BSMV. Light blue, outer chloroplast membrane (OM); translucent white, inner chloroplast membrane (IM); light yellow, spherules derived from the outer membrane. **(D)** Representative tomogram slice of remodeled chloroplast in BSMV-infected *N. benthamiana* leaves by FIB-SEM. The arrowhead indicates the opening of a CI. **(E)** 3D visualization by FIB-SEM of a chloroplast in a BSMV-infected cell. Chl, chloroplast; CI, cytoplasmic invagination; green, chloroplast; yellow, CI; white arrowheads, chloroplast invaginations; black arrow, CI aperture. The inset shows an enlarged view of a CI. This figure is adapted with permission from Jin et al. (2018) (© 2018 by the American Society of Plant Biologists).

Barley stripe mosaic virus (BSMV) is the type member of the genus *Hordeivirus* in the family *Virgaviridae* (Jackson et al., 2009; Adams et al., 2017). The (+) strand RNA genome of BSMV encodes two replication proteins, αa and γa, which are localized in chloroplasts (Zhang et al., 2017). In BSMV-infected plants, the membranous structures of the chloroplasts change dramatically (Carroll, 1970; Lin and Langenberg, 1985; Torrance et al., 2006). Peripheral invaginations (Figures 5A,B) and large cytoplasmic invaginations (CIs) containing abundant virus-like particles (VLPs) are observed within the chloroplasts. Around the CI, similar invaginations are observed in which small spherules are occasionally observed (Figure 5A; Jin et al., 2018). Immunoelectron microscopy (IEM) indicates that viral dsRNA and the replication protein αa are specifically enriched in the invaginations at the periphery of the chloroplast envelope and the CIs, suggesting that BSMV replicates in these invaginations. ET was recently used to characterize the 3D architecture of chloroplast remodeling during BSMV infection. The generated model reveals that invaginations containing one or more spherules are formed from the chloroplast inner membrane (Figures 5A,B). These spherules, which have an internal diameter of ~50 nm, are generated from invaginations of the chloroplast outer membrane (Figure 5C). Each spherule has a neck that extends toward the cytoplasm, suggesting that these spherules are the true sites of BSMV replication (Figure 5C; Jin et al., 2018). The spherules around the CI also have necks that connect to the CI lumen, and IEM using serum containing antibodies against CP indicates that the virus-like particles (VLPs) inside the CI are BSMV virions. Thus, CI may be associated with the assembly of virus particles, and the existence of replicative spherules around the CI argues that the replication and assembly of BSMV are coupled processes. Furthermore, it was shown by FIB-SEM that the chloroplasts in BSMV-infected cells are extensively remodeled, as manifested by cavern-like invaginations with protrusions on their surfaces, and that CIs are surrounded by a double membrane and display irregular shapes with apertures of various sizes (Figures 5D,E). The replicative spherules of BSMV that are found between the chloroplast inner and outer membranes resemble the replication factories built by FHV (Kopek et al., 2007), and all of these spherules have openings to the cytoplasm. The CIs connected to the cytoplasm (Figures 5D,E) appear somewhat similar to the dilations within mitochondria induced by MNSV (Figures 3A,B), as evidenced by their outward connections and by the presence of peripheral spherules/vesicles.

In summary, despite having somewhat different morphologies, the replication factories built by plant (+) RNA viruses belonging to different groups have many similarities, as do the replication factories produced by plant and animal viruses. In plants, the invaginated spherules/vesicles formed by negative membrane curvature are usually 50–150 nm in diameter and have neck-like connections to the cytoplasm. Like animal viruses, SMT and DMT structures induced by plant (+) RNA viruses are highly motile and morphologically dynamic, implying that viral replication is tightly linked to particular stages of the viral infection cycle. Characterization of the 3D architectures of various membranous VRCs thus broadens our knowledge of the cellular structures that are formed during virus-host interaction and provides structural insight into the replication factories of (+) RNA viruses. We also note that most of the currently available 3D tomograms of plant virus replication factories were generated from chemically fixed samples (Cao et al., 2015; Gómezaix et al., 2015; Fernández de Castro et al., 2017; Jin et al., 2018); such fixation may induce ultrastructural artifacts due to the slow diffusion of chemical fixatives and to selective cross-linking by chemical fixatives (Gilkey and Staehelin, 1986). Therefore, preparation of samples using cryo-methods should enable better preservation of the native structure of the specimen and is expected to display the detailed ultrastructure of virus-infected plant cells more accurately.

## Biogenesis of viral replication compartments

Virus-orchestrated membrane alterations leading to the production of replication factories depend on the action of one or more viral proteins (Paul and Bartenschlager, 2013; Laliberté and Zheng, 2014; Kovalev et al., 2016). These critical proteins are usually integral membrane proteins. They localize to a given organelle through their location signals or interact with membrane proteins or lipids and then initiate membrane bending. Although expression of these proteins alone may induce the formation of altered membranous structures similar to those found in virus-infected cells (Schwartz et al., 2002), in some cases the remodeled membranes induced by these proteins in the absence of virus differ from those observed during infection (Cao et al., 2015). Viral replication proteins are the major contributors to the alteration of endomembranes that occurs during virus infection. By localizing to the membrane, they also recruit other viral and host proteins or RNAs to the replication sites, leading to membrane deformation. The dynamic bending of the membranes is achieved mainly by the asymmetric interactions of proteins with the membrane and by local alterations in membrane lipid composition (McMahon and Gallop, 2005). The insertion of replication proteins into the membrane depends on their transmembrane domains or their amphipathic helices, and sometimes self-interactions and interactions with lipids are equally important for generating and stabilizing membrane curvature. Moreover, the remodeling of the membrane involves diverse host factors, including membrane-shaping proteins and components of the early secretory pathway. We list some typical plant viral proteins and host factors that directly function in cellular remodeling in Table 1; below, we discuss some viruses that have been intensively studied in terms of the viral and host factors that are required for the production of their replication factories.

**Table 1:** Viral and host factors involved in endomembrane remodeling during the establishment of plant (+) RNA viral replication factories.

**Family**	**Genus**	**Virus**	**Type of membrane structures**	**Viral factors**	**Membrane source**	**Host factors**	**References**
*Potyviridae*	*Potyvirus*	*Turnip mosaic virus Tobacco etch virus*	VesiclesVesicles	6K_2_6K_2_	ERER	Sec24, Sar1, Arf1, SNARESec24, Sar1, Arf1	Schaad et al., 1997; Beauchemin et al., 2007; Wei and Wang, 2008; Grangeon et al., 2012; Wei et al., 2013; Jiang et al., 2015
	*Bymovirus*	*Wheat yellow mosaic virus*	Membranous inclusion bodies	p2	ER	Sar1	Sun et al., 2014
*Bromoviridae*	*Bromovirus*	*Brome mosaic virus*	Spherules	1a	ER	ESCRT-III, Vps4AAA+ ATPaes, reticulon, ACB1, Cho2p, Erv14, Sec24, Sec13, Sec31, PC	Schwartz et al., 2002; Menzel et al., 2009; Diaz et al., 2010, 2012, 2015; Diaz and Ahlquist, 2012
*Secoviridae*	*Comovirus*	*Cowpea mosaic virus*	Vesicles	Co-Pro, NTB-VPg	ER	*de novo* lipid synthesis	Carette et al., 2000
	*Nepovirus*	*Tomato ringspot virus*	–	NTP-VPg, X2	ER	–	Han and Sanfacon, 2003; Zhang and Sanfaçon, 2006
		*Grapevine fanleaf virus*	Vesicles	VPg	ER	–	Ritzenthaler et al., 2002
*Virgaviridae*	*Pecluvirus*	*Peanut clump virus*	MVBs, vesicles	p131, p191	ER	–	Dunoyer et al., 2002
	*Tobamovirus*	*Tobacco mosaic virus*	–	p126, MP	ER	TOM1, TOM2, TOM3, Actin and myosin, Microtubule, PAP85	Heinlein et al., 1998; Reichel and Beachy, 1998; Yamanaka et al., 2000, 2002; Hagiwara et al., 2003; Tsujimoto et al., 2003; Kawakami et al., 2004; Liu et al., 2005; Chen et al., 2013; Heinlein, 2015
		*Tomato mosaic virus*	–	p126	ER	TOM1, TOM3	Yamanaka et al., 2000, 2002; Nishikiori et al., 2006
*Tombusviridae*	*Betanecrovirus*	*Beet black scorch virus*	Spherules	p23	ER	–	Cao et al., 2015
	*Dianthovirus*	*Red clover necrotic mosaic virus*	–	p27	ER	PLD, PA, Arf1, Sar1	(Turner et al., 2004; Hyodo et al., 2013, 2015;)
	*Aureusvirus*	*Cucumber leaf spot virus*	–	p25	ER	–	Ghoshal et al., 2014
	*Carmovirus*	*Melon necrotic spot virus*	MVBs	p29	Mitochondria	–	Mochizuki et al., 2009
	*Tombusvirus*	*Carnation Italian ringspot virus*	MVBs	p36	Mitochondria	ESCRT-I, Rab5 small GTPase, PE	Burgyan et al., 1996; Hwang et al., 2008; Richardson et al., 2014; Xu and Nagy, 2016
		*Tomato bushy stunt virus*	MVBs	p33, RNA	Peroxisome	ESCRT-I, ESCRT-III, Bro1p, Vps4AAA+ ATPaes, ORPs, VAPs, Ino2, Ino4, Rab5 small GTPase, sterol, PE, PC, Actin	McCartney et al., 2005; Jiang et al., 2006; Barajas et al., 2009, 2014a,b,c; Sharma et al., 2010, 2011; Xu and Nagy, 2015, 2017; Kovalev et al., 2016; Nagy, 2016; Nawaz-Ul-Rehman et al., 2016;
		*Cymbidium ringspot virus*	MVBs	p33	peroxisome	–	Burgyan et al., 1996; Navarro et al., 2004
		*Cucumber necrosis virus*	MVBs	p33	Peroxisome	PE	Rochon et al., 2014; Xu and Nagy, 2015
*Tymoviridae*	*Tymovirus*	*Turnip yellow mosaic virus*	Vesicles	66K	Chloroplast	–	Prod'homme et al., 2001
*Virgaviridae*	*Hordeivirus*	*Barley stripe mosaic virus*	Spherules	αa	Chloroplast	–	Jin et al., 2018

*“—” data not available*.

### Brome mosaic virus (BMV)

BMV is one of the best-characterized (+) RNA viruses in terms of its replication (Diaz and Wang, 2014). In plants, BMV replication is highly associated with the ER (Restrepo-Hartwig and Ahlquist, 1996). In yeast, expression of BMV replication proteins 1a and 2a and of RNA3 derivatives can also support BMV RNA replication (Janda and Ahlquist, 1993; Ishikawa et al., 1997); as in the plant host systems, proteins 1a and 2a target the ER for RNA synthesis, thereby duplicating the major features of BMV replication in plant cells (Restrepo-Hartwig and Ahlquist, 1999). Hence, yeast cells must contain the necessary factors for BMV replication. In plant cells, BMV infection induces the formation of ER-derived vesicular structures (Schwartz et al., 2002; Bamunusinghe et al., 2011). In yeast cells, BMV-encoded replication protein 1a alone is capable of inducing the formation of ER-derived spherules associated with RNA synthesis. These ER-derived membranous structures are similar in appearance to those observed in BMV-infected plant cells (Schwartz et al., 2002). 1a has no transmembrane domain but instead contains an amphipathic α-helix, helix A, that is crucial for the protein's association with and subsequent rearrangement of membranes. In yeast, mutations within helix A lead to two distinct phenotypes that are characterized by the absence of membrane invaginations and the formation of abundant but smaller-sized spherules (Liu et al., 2009). Moreover, 1a self-interactions involving the RNA capping and helicase domains are critical for the protein's association with the ER membrane and its ability to induce the formation of spherules (Liu et al., 2009; Diaz et al., 2012). Hence, when helix A inserts into the membrane, intramolecular and intermolecular interactions cause hundreds of 1a monomers to form an inner shell that induces the formation of 50–70 nm spherules. This is consistent with the high copy number of 1a molecules in a spherule. Replication protein 2a also helps determine the conformation of BMV replication factories. Modulation of the relative expression levels of replication proteins 1a and 2a or disturbance of the interactions between them alters the morphology of the viral replication factories (Schwartz et al., 2004; Bamunusinghe et al., 2011).

The 1a protein also recruits some host factors that help increase the membrane curvature. Endosomal sorting complexes required for transport (ESCRT) proteins participate in sorting cargo proteins from the endosomes to MVBs through membrane invagination and vesicle formation; this process requires the sequential recruitment of ESCRT-0, ESCRT-I, II, III, and Vps4p, which generate membrane invaginations and subsequently direct membrane budding and scission (Wollert et al., 2009; Wollert and Hurley, 2010; Alonso et al., 2016). The production of BMV and of many other (+) RNA viruses, including the TBSV-induced spherules that will be discussed below, shares some topological similarities with the formation of ESCRT-dependent MVB vesicles and the budding of enveloped retroviruses. These replicative spherules are not released from the membrane and remain connected to it via a neck-like opening. The ESCRT-III effector Snf7p interacts strongly with BMV 1a and is recruited by 1a to sites of viral replication. Deletion of Snf7p abolishes spherule formation and inhibits BMV replication, and deletion of other ESCRT-III factors such as Vps20p, Vps24p, and Vps2p modulates the number of spherules that are produced (Diaz et al., 2015). ESCRT-III factors have been proposed to function coordinately with cargo proteins in the limiting membranes, leading to membrane invagination. Although the interaction between 1a and ESCRT-III may facilitate 1a-induced membrane remodeling, it is not known how the replicative spherules avoid being released from the ER membrane or how they form closed vesicles. Another class of membrane-shaping proteins, reticulon homology proteins (RHPs), is needed for the formation of BMVs in the replication factory. RHPs comprise a family of membrane-shaping proteins that function in the formation and stabilization of curved peripheral ER tubules (De Craene et al., 2006; Voeltz et al., 2006; Wakefield and Tear, 2006; Tolley et al., 2008); they are redistributed from ER tubules to BMV-induced replication compartments through interaction with BMV 1a (Diaz et al., 2010; Diaz and Ahlquist, 2012). EM analysis has indicated that complete deletion of RHPs abolishes spherule formation and viral replication and that their partial depletion results in the production of smaller-sized spherules. The fact that RHP participates in the formation of nuclear pores and modulates the size of the spherules suggests that it has a role in stabilizing the necks of the spherules (Diaz et al., 2010; Diaz and Ahlquist, 2012). Coat protein complex I (COPI) and COPII vesicles within the early secretory pathway for transporting proteins and lipids are also hijacked by BMV for 1a distribution (Beck et al., 2009; Brandizzi and Barlowe, 2013). A cargo receptor of COPII vesicles, the 14-kDa ER-vesicle protein Erv14, and the COPII coat component Sec24 are required for the recycling of BMV 1a from peripheral tubular ER to the perinuclear ER membrane. Deletion of Erv14 leads to decreased numbers of spherules and larger spherule size (Li et al., 2016), suggesting that the interactions between 1a, Erv14 and Sec24 may facilitate the enrichment and self-interaction of 1a, a necessary process for membrane remodeling and for the stabilization of spherules.

Replication factory membrane scaffolds are enriched in specific lipids that may be derived from existing lipid sources or synthesized *de novo*. Hence, viruses usually modulate host lipid metabolism in a way that favors the formation of replication factories. BMV infection is accompanied by the accumulation of lipids, indicating that these lipids participate directly or indirectly in BMV replication (Lee and Ahlquist, 2003). Recent investigations have shown that phosphatidylcholine (PC) accumulates at BMV replication sites though recruitment of the PC synthesis machinery by the 1a protein (Zhang et al., 2016). In addition, ACB1-encoded acyl coenzyme A (acyl-CoA) binding protein (ACBP), a protein that promotes general lipid synthesis, is required for the assembly of replication factories (Zhang et al., 2012). Blocking PC synthesis leads to the formation of spherules with larger diameters, whereas deletion of ACBP leads to the production of a larger number of smaller spherules (Zhang et al., 2012, 2016). The morphology of the smaller spherules is similar to that of the spherules induced by the 1a mutant, suggesting that the formation of appropriate spherules requires interaction between 1a and lipids. Deletion of lipid synthesis genes or blockage of PC synthesis may lead to insufficient production of lipids for the formation of replication factories. Altered membrane lipid composition might further affect interactions between 1a and membranes; because hundreds of 1a proteins associate with the spherule's inner membrane to form a shell-like structure, the interaction of 1a with membrane lipids may regulate spherule size.

### Viruses in *Tombusviridae*

The replication proteins of viruses in the *Tombusviridae* usually have a targeting signal; when expressed alone, these proteins can induce specific organelle membrane remodeling (Burgyan et al., 1996; Rubino and Russo, 1998; Navarro et al., 2004; McCartney et al., 2005; Panavas et al., 2005; Hwang et al., 2008; Mochizuki et al., 2009; Rochon et al., 2014; Gómezaix et al., 2015). Viral genomic RNAs also participate in the formation of the replication factory. Recent studies of tombusviruses have shown that spherule size correlates with the length of the viral RNA template (Kovalev et al., 2016). Similar results have also been reported for other animal viruses such as SFV and FHV (Kallio et al., 2013; Ertel et al., 2017). It is worthy of note that a recent cryo-ET study of FHV replication spherules revealed a novel crown-like structure surrounding the spherule's necked aperture (Ertel et al., 2017). This observation provides new insight into the export of viral progeny RNA from the spherules.

Replication proteins also recruit host factors that remodel membranes during the assembly of replication factories. Like BMV replication, TBSV replication can be reconstituted in yeast. Previous studies indicated that TBSV defective interfering (DI) RNA can replicate in yeast cells (Panavas and Nagy, 2003), and a cell-free system based on yeast supports the replication of full-length TBSV genomic RNA (Pogany and Nagy, 2008). In plant and yeast cells, both TBSV infection and repRNA replication induce the formation of peroxisome-derived MVBs; these spherule-like structures have necks that contact the peroxisome boundary membrane (McCartney et al., 2005; Barajas et al., 2014b; Fernández de Castro et al., 2017), suggesting evolutionary conservation of the selection of replication sites and VRC structures across different kingdoms. Hence, yeast has been developed as a surrogate model host for the study of TBSV replication and used to screen for host factors involved in TBSV replication (Nagy, 2008). Genome-wide screening using the TBSV-yeast model system indicates that seven ESCRT proteins are involved in the replication of TBSV (Jiang et al., 2006). TBSV recruits ESCRT proteins for membrane remodeling. Ubiquitinated TBSV p33 protein interacts with ESCRT-I Vps23p and its accessory ESCRT factor Bro1p and then sequentially recruits the ESCRT-III machinery and Vps4 AAA+ ATPase to the replication sites to induce the formation of spherule-like structures (Li et al., 2008; Barajas et al., 2009, 2014a; Barajas and Nagy, 2010; Imura et al., 2015). Vps4 has also been identified as a component of the TBSV replication complex, and EM analysis of yeast cells infected by TBSV indicated that deletion of Vsp4 leads to the formation of crescent-like membrane structures that lack neck-like openings (Barajas et al., 2014a; Kovalev et al., 2016). Thus, a non-canonical role of Vps4 would be facilitation of the stabilization of neck structures of the TBSV-induced spherules and prevention of membrane scission. Similar results have also been reported for other tombusviruses. For instance, CIRV recruits ESCRT-I protein to mitochondrion-derived VRCs through the p36 replication protein (Richardson et al., 2014). In addition, ESCRT factors also participate in the formation of BMV-induced spherules, as discussed above (Diaz et al., 2015). Hence, ESCRT factors are evolutionarily conserved host factors that are used by different viruses to promote spherule formation and to stabilize the neck-like structures of spherules.

Similar to the process that occurs during BMV infection, tombusvirus-induced membrane deformation requires lipid synthesis to support the huge proliferation of ER and peroxisome membranes (Sharma et al., 2011; Barajas et al., 2014c). Furthermore, sterols and phospholipids are enriched in the cellular locations at which TBSV RNA replication occurs in both plant cells and yeast (Sharma et al., 2010; Xu and Nagy, 2015, 2017). Sterols are essential membrane components that determine the curvature and fluidity of membranes (Lorizate and Kräusslich, 2011). The replication proteins p33 and p92 bind directly to sterols *in vitro* (Xu and Nagy, 2017). In addition, TBSV p33 interacts with oxysterol-binding ORP and VAMP-associated proteins (VAP), which mediate the redistribution of sterols to viral replication sites and facilitate the bending of membranes (Barajas et al., 2014b). The enrichment of sterols at viral replication sites in yeast cells may facilitate the efficient sequestration of replication proteins and may affect the topologies and structures of the replication proteins that remodel membranes. Large sterols might also maintain the stability of spherules for a longer time than that provided by ESCRTs. Phosphatidylethanolamine (PE) is the most abundant class of phospholipid at TBSV replication sites in yeast cells. Increases in PE levels as well as in phosphatidylcholine (PC) levels enhance TBSV RdRp activity, whereas increased phosphatidylglycerol (PG) levels have an inhibitory effect on TBSV replication (Xu and Nagy, 2015). Other tombusviruses, including cucumber necrosis tombusvirus (CNV) and CIRV, similarly induce the enrichment of PE in VRCs (Xu and Nagy, 2015). Further studies have shown that the enrichment of PE in TBSV replication factories is mediated by the direct interaction of the replication protein p33 with the endosomal Rab5 small GTPase, which leads to the redistribution of PE to viral replication sites (Xu and Nagy, 2016). PE is a cone-shaped lipid with a hydrophilic head and with a hydrophobic tail of varying length, and its enrichment can induce membrane curvature. The recruitment of PE to replication sites by p33 could facilitate membrane proliferation and spherule formation. The results of recent METTEM and 3D molecular mapping studies of TBSV-induced spherules in yeast cells show that the localization of p33 to an appropriate environment in which specific cofactors and lipids are present is a crucial step in viral replication (Fernández de Castro et al., 2017), substantiating the role of PE in supporting viral replication.

The cytoskeleton and motor proteins also play important roles in the formation and anchorage of viral replication complexes in plants. For example, recent studies indicate that the actin network is targeted by TBSV to support its replication. Confocal microscopy analysis showed that actin patches are closely associated with large p33-containing replication organelle-like structures in yeast cells and that such patches are present throughout the large replication compartments in plant cells, suggesting that actin plays a role in recruiting viral and cellular components (e.g., lipid) for VRC assembly (Nawaz-Ul-Rehman et al., 2016; Xu and Nagy, 2016). In addition, the formation and movement of TMV VRCs requires the involvement of the cytoskeleton (Heinlein et al., 1998; Kawakami et al., 2004; Liu et al., 2005). TMV 126-kD protein regulates VRC size and facilitates the movement of VRCs along microfilaments (Liu et al., 2005). TMV-induced VRCs also contain viral movement protein (MP), which is targeted to the junctions of microtubules and ER (Martelli and Russo, 1977; Ashby et al., 2006; Sambade et al., 2008; Peiró et al., 2014). Furthermore, expression of MP alone leads to ER aggregation (Reichel and Beachy, 1998). The close association of MP with microtubules and ER strongly suggests that it plays a role in the formation of VRCs (Heinlein, 2015). Additional electron tomography of virus replication factories and the cytoskeletal network should be performed to determine their spatial relationships at high resolution and in three dimensions.

Red clover necrotic mosaic virus (RCNMV), another virus in *Tombusviridae*, promotes ER rearrangement for replication by inserting viral p27 into the membranes. Moreover, RCNMV hijacks the ADP ribosylation factor 1 (Arf1), a small GTPase involved in the formation of COPI vesicles within the early secretion pathway (Beck et al., 2009), to the viral replication sites (Hyodo et al., 2013). Dimerization of Arf1 is critical for positive membrane curvature during formation of coated vesicles (Beck et al., 2008; Krauss et al., 2008). The perturbation of ER morphology by p27 may depend on the interaction of p27 with Arf1 as well as its ability to bend membranes.

### Viruses in *Potyviridae*

The 6K_2_ protein of tobacco etch virus (TEV) and TuMV is an integral membrane protein that induces the formation of ER-derived vesicles during infection (Schaad et al., 1997; Beauchemin et al., 2007; Cotton et al., 2009). Early secretory pathways have been found to play an extensive role in potyviral infection. Biogenesis of 6K_2_ vesicles at ERES relies on the COPI and COPII machinery (Wei and Wang, 2008), and EM analysis showed a close association of bulging membranes and vesicles with the ER (Grangeon et al., 2012). Further studies indicated that the 6K_2_ protein of TuMV interacts with the COPII coatomer Sec24a protein (Jiang et al., 2015) and that this interaction modifies the ER-Golgi interface and disrupts the protein secretion pathway. This block in protein and lipid transport between ER and Golgi may lead to the membrane aggregation and proliferation that is observed by EM (Wan et al., 2015). Functional disruption of Sec24a, Sar1, or the COPI component Arf1 protein compromises the formation of 6K_2_ vesicles (Wei and Wang, 2008), supporting their role in membrane remodeling. The COPII protein Sar1 also interacts with the wheat yellow mosaic virus (WYMV) p2 protein, resulting in rearrangement of the ER during WYMV infection (Sun et al., 2014). Interestingly, the Golgi apparatus appears not to be affected during virus infection, suggesting that a Golgi bypass is possible. Recent studies have shown that unconventional secretory pathways are involved in the formation of MVBs (Ding et al., 2012).

The examples given above provide a good illustration of a common theme in membrane remodeling and the formation of spherules/vesicles: viral proteins, sometimes with the involvement of viral RNAs, recruit host factors that are diverted from their original functions and used to create virus replication factories (Diaz and Wang, 2014; Laliberté and Zheng, 2014; Wang, 2015; Nagy, 2016). However, further studies, especially studies of how these interactions correlate with and are mirrored by the 3D architecture of virus replication factories, are required. All of the 3D reconstructions of plant virus replication factories reported to date have been derived from wild-type virus-infected cells. However, little information regarding the 3D structure of the aberrant replication factories that are produced in response to the disruption of these interactions is available. In addition, the 3D spatial relationships between host factors and virus-induced membrane structures remain to be fully characterized.

## Conclusions and future research

(+) RNA viruses replicate their genomes in association with the remodeling of cytoplasmic membranes. Advanced 3D imaging techniques enable the visualization and analysis of membrane morphologies in three dimensions. To date, 3D structures of the replication organelles of several plant viruses have been proposed, providing deeper insight into the assembly and biogenesis of virus replication factories. More and more host factors, such as the membrane-shaping and lipid synthesis-related proteins, have been shown to be involved in the formation of membranous replication factories. The results of these studies improve our understanding of the replication machinery in membrane compartments and provide new potential targets for the design of antiviral strategies.

Despite these recent advances, our current knowledge of the 3D architecture of (+) RNA virus replication factories is largely descriptive. There are still many issues that need to be addressed. For example, the dynamic changes that occur during virus-induced cellular remodeling, especially how viral and cellular components are transported into and out of replication factories, have not been thoroughly elucidated to date. A combination of live-cell imaging, correlative light-electron microscopy, and new techniques for molecular probing would be helpful in determining the structural and dynamic aspects of viral replication factory biogenesis and its regulation in live cells and would be particularly useful for determining how cellular components interact with the virus to spatially and temporally regulate membrane remodeling during virus infection. It is fascinating to see how the 3D architectures of virus replication factories derived from different plant viruses or under various exogenous conditions directly reflect the molecular processes underlying viral replication. In addition, due to the tight coupling of viral replication with translation and viral particle assembly, how viruses utilize cellular membranous compartments to spatiotemporally coordinate different stages in the viral life cycle needs to be further addressed in the future. High-throughput profiling of purified viral replication factories should make it possible to determine their protein and lipid content, thereby providing a full view of the host constituents of viral replication factories and making it possible to decipher the mechanisms underlying virus-induced cellular remodeling. At the same time, virus-induced membrane curvature increases the surface area of the host endomembranes, which increases the likelihood of contact between different organelles. Recent studies have indicated that membrane contact sites (MCSs) play an important role in the communication between various organelles; this particularly applies to the ER, which contacts almost all other cellular organelles (Helle et al., 2013). It will be interesting to determine the function of MCSs in mediating the transport of host molecules to viral replication sites and how MCSs function in the perception of viral assault by the plant and thereby cause it to launch an antiviral response.

## Author contributions

XJ, YZ, XC, XW, JJ, and JW wrote the manuscript. YZ and J-FL designed and revised the manuscript.

### Conflict of interest statement

The authors declare that the research was conducted in the absence of any commercial or financial relationships that could be construed as a potential conflict of interest.
